# ^18^F-FDG uptake for prediction EGFR mutation status in non-small cell lung cancer

**DOI:** 10.1097/MD.0000000000004421

**Published:** 2016-07-29

**Authors:** Jian Guan, Nan J. Xiao, Min Chen, Wen L. Zhou, Yao W. Zhang, Shuang Wang, Yong M. Dai, Lu Li, Yue Zhang, Qin Y. Li, Xiang Z. Li, Mi Yang, Hu B. Wu, Long H. Chen, Lai Y. Liu

**Affiliations:** a Department of Radiation Oncology; b PET Center; c Department of Pathology; d Department of Respiratory and Critical Care Medicine, Chronic Airways Diseases Laboratory, Nanfang Hospital, Southern Medical University, Guangzhou, Guangdong, China.

**Keywords:** EGFR, NSCLC, PET/CT, prediction model, prospective validation

## Abstract

Epidermal growth factor receptor (EGFR) mutations in non-small cell lung cancer (NSCLC) are a response to EGFR-tyrosine kinase inhibitor. However, a lack of sufficient tumor tissue has been a limitation for determining EGFR mutation status in clinical practice. The objective of this study was to predict EGFR mutation status in NSCLC patients based on a model including maximum standardized uptake value (SUVmax) and clinical features.

We retrospectively reviewed NSCLC patients undergoing EGFR mutation testing and pretreatment positron emission tomography/computed tomography between March 2009 and December 2013. The relationships of EGFR mutations with both SUVmax and patient characteristics were evaluated, and a multivariate logistic regression analysis was performed. The model was assessed by area under the receiver-operating characteristic curve (AUC) and was prospectively validated during January to June 2014.

Three hundred and sixteen patients meeting the criteria were enrolled for model construction. The SUVmax values were significantly lower for EGFR mutations (mean, 9.5 ± 5.74) than for EGFR wild-type (mean, 12.7 ± 6.43; *P* < 0.001). ROC curve analysis showed that the SUVmax cutoff point was 8.1, for which the AUC was 0.65 (95% confidence interval [CI], 0.60–0.72). In addition, multivariate analysis also showed that low SUVmax (≤8.1) was a predictor of EGFR mutations, for which the AUC was 0.77, combining nonsmoking history and primary tumor size (≤5 cm). Eighty-five patients were enrolled to validate the predictive model, and the overall accuracy, sensitivity, and specificity were 77.6%, 64.6% (95% CI 40.7–82.8), and 82.5% (95% CI 70.9–91.0), respectively.

The specific FDG uptake value could be considered to effectively predict EGFR mutation status of NSCLC patients by considering smoking history and primary tumor size when genetic tests are not available.

## Abstract 2

Supplemental Digital Content is available in the text

## 1 Introduction

Lung cancer is one of the main contributors to cancer-related deaths worldwide.^[1]^ The treatment of non-small cell lung cancer (NSCLC) has seen great advances in the last decade. The epidermal growth factor receptor (EGFR) tyrosine kinase inhibitor (TKI) has a good effect as a treatment for NSCLC cases with activated EGFR mutations.^[2,3]^ Compared with standard chemotherapy, upfront TKI has a better progression-free survival in patients with EGFR mutations.^[4–6]^ However, a lack of sufficient tumor tissue has an impact on genetic testing in clinical practice.

Fluorine-18 fluorodeoxyglucose positron emission tomography/computed tomography (FDG PET/CT), which is connected to high glucose metabolism, plays an important role in initial staging, restaging after therapy, and radiation therapy planning.^[7–9]^ Moreover, in the clinical practice, early decreases in FDG uptake are predictive of efficacy of TKI.^[10–12]^ Therefore, as a noninvasive method, quantification of glucose metabolism with FDG-PET is one way to predict EGFR mutations.

The prediction model built in this study was used to predict the EGFR mutation status of NSCLC patients. We carried out a retrospective study in patients who underwent EGFR mutation testing and pretreatment with FDG-PET/CT in NSCLC, and we discuss the relationships between ^18^F-FDG uptake, clinical features, and EGFR mutation status. We then establish a predictive model for EGFR mutation status, and we validate the model by using prospective samples.

## 2 Materials and methods

### 2.1 Patients

We retrospectively investigated the medical records of patients treated between March 2009 and December 2013. All of them met the following entry criteria: diagnosis was made either histologically or cytologically, and the patients underwent *EGFR* gene testing; PET/CT was performed previous to any therapy; histopathology was reviewed at Nan Fang Hospital in Guangzhou.

Eighty-five patients from January through June 2014 were enrolled and were used to prospectively validate the prediction model. The parameters were collected as before. The EGFR mutation analysis was performed blinded to predicted outcome data. The study was conducted with the approval of the Nan Fang Hospital Institutional Review Board.

All procedures performed in studies involving human participants were in accordance with the ethical standards of the institutional and/or national research committee and with the 1964 Helsinki declaration and its later amendments or comparable ethical standards.

### 2.2 EGFR mutation analysis

EGFR mutations in exons 18, 19, 20, and 21 were detected by direct sequencing in the pathology department of Nan Fang Hospital in 2009–2012. Genomic DNA was collected from tumor specimens using the TaKaRa DEXPATTM Kit (TaKaRa), and the EGFR reference sequence was acquired from the NCBI database. Genomic DNA sequences were obtained by polymerase chain reaction (PCR)-based direct sequencing. After that, pure PCR products were sequenced in both the forward and reverse directions using the ABI PRISM BigDye Terminator Cycle Sequencing Ready Reaction kit (Version 3) and an ABI PRISM 3730XL Genetic Analyzer (Applied Biosystems, Carlsbad, CA). The chromatograms were analyzed by an experienced reviewer.

The Amplification Refractory Mutation System (ARMS) was used to detect exons 18 to 21 in 2013. All ARMS primer pairs were used for PCR with the following criteria: concentration of 1 μmol/L, control reaction primers at concentration 0.1 μmol/L, and TaqMan probes at concentration 0.5 μmol/L. Cell line DNA sequences were amplified by PCR. After PCR, the cycle threshold values of the target gene and reference genes were obtained by DxS (Manchester).

### 2.3 Covariates

Patient characteristics including age, sex, and smoking status were recorded before treatment. Smoking status was divided into 2 groups: nonsmokers who never had cigarettes in their lifetime, smokers who were smoking for a period before diagnosis. Tumor characteristics such as histology, grade, and stage were recorded. These tumor characteristics were collected from the clinical pathology reports, and the stage was specified according to the seventh edition of the American Joint Committee on Cancer Staging Manual (AJCC).

### 2.4 FDG-PET/CT

The fused PET/CT, PET, and CT images were independently assessed by 2 experienced nuclear medicine physicians. Original focus was identified by a visual qualitative analysis. A volume of interest was placed over the primary tumor to quantify the uptake. The maximum voxel uptake, reflecting the maximal uptake of FDG within the tumor, was found and its maximum standardized uptake value (SUVmax) was calculated according to the following formula: SUV = tissue radioactivity concentration (becquerels per milliliter)/(injected dose\[becquerels]/patient weight [grams]). For patients with >1 primary lesion, the SUVmax was calculated for all primary lesions as above. Then, the largest SUVmax was selected for analysis.^[13]^ Patients received treatment 1 month after the FDG PET scan.

### 2.5 Statistical analysis

Continuous covariates were analyzed using Student *t* test or Wilcoxon rank-sum test. Categorical covariates were analyzed with the Pearson *χ*^2^ test or Fisher exact test as appropriate. A receiver-operating characteristic (ROC) curve was generated to determine a cutoff for the SUVmax of the primary tumor. Multivariate logistic regression analysis was performed to test the variables that yielded the predictors of EGFR mutations. The area under the ROC curve (AUC) was used for the predictive value. All *P* values <0.05, which were derived from 2-sided tests, were considered significant. SPSS (Version 21.0; SPSS Incorporation, Chicago, IL) was used for the analysis.

## 3 Results

### 3.1 Clinical features and EGFR mutations

**Figure 1:**
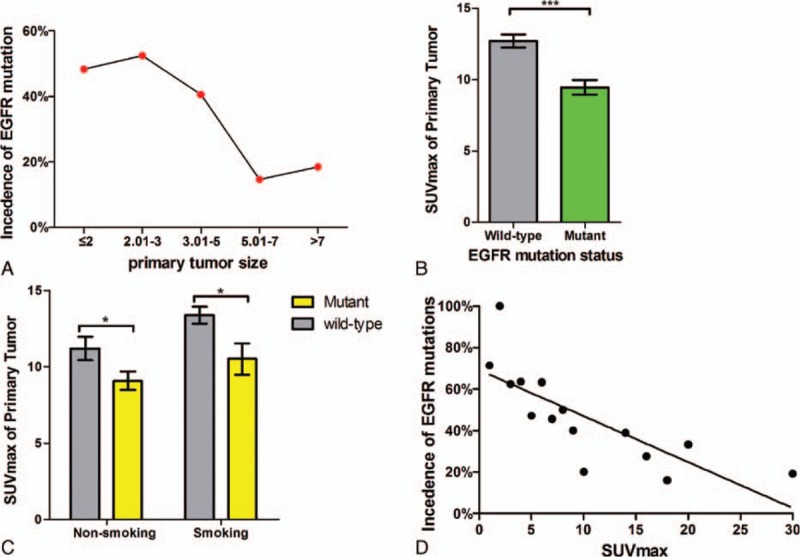
(A) Incidence of EGFR mutations according to primary tumor size. (B) Box plot of SUVmax of primary tumor, with mean value indicated. (C) SUVmax of different EGFR mutation status in patients with different smoking history. (D) Incidence of EGFR mutations according to the SUVmax. Data are represented as the mean ± SEM. ^∗^*P* < 0.001, ^∗∗^*P* > 0.05. EGFR = epidermal growth factor receptor, SUVmax = maximum standardized uptake value.

The baseline characteristics of the patients are listed in Table 1. There were 316 patients (216 males and 100 females) that met the eligibility criteria (Supplemental Digital Content 1). Of those, 126 patients (39.9%) were EGFR mutation-positive. The EGFR mutations were more frequent in female patients than males (64.0% vs. 28.7%, *P* < 0.001). In these patients, 2, 63, 6, 45, and 10 patients had exon 18, 19, 20, and 21 mutations and multipoint mutations, respectively. The median age was 60 years, and 162 patients (51.3%) had a history of smoking. The EGFR mutations of nonsmokers were more frequent than those of smokers (60.4% vs. 20.4%; *P* < 0.001). There were 242 patients (76.6%) with adenocarcinoma. EGFR mutations were more frequent in adenocarcinoma (*P* < 0.001). Most of the patients, whose CT findings revealed a primary tumor size >5 cm, had wild-type EGFR (83.8%; *P* < 0.001). When primary tumor size was classified in 5 groups (≤2, 2.01–3, 3.01–5, 5.01–7, and >7), there was a trend of lower incidence of EGFR mutations in larger tumors (48.3%, 52.4%, and 40.6%, 14.6%, 18.5%, respectively; *P* < 0.001) (Fig. 1A).

**Table 1:**
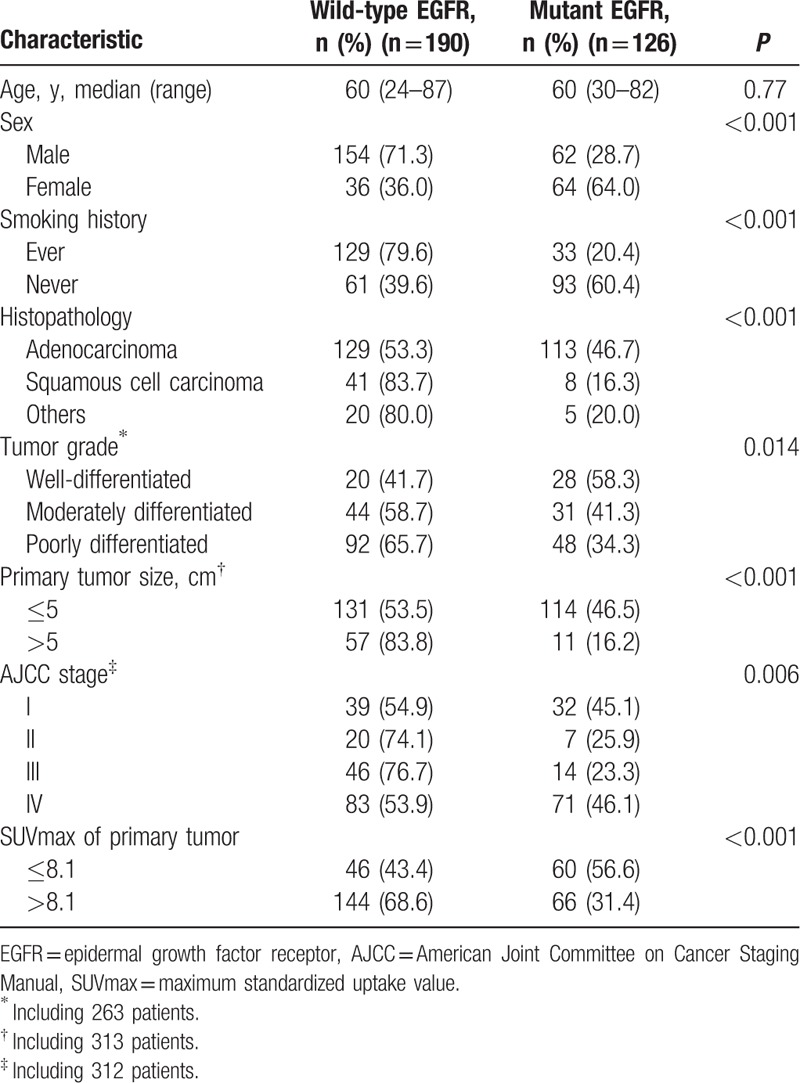
Association of clinical feature and EGFR mutation.

### 3.2 Association of SUVmax and EGFR mutations

The SUVmax was significantly lower in cases of EGFR mutations (mean, 9.5 ± 5.74) than in cases of wild-type EGFR (*P* < 0.001; mean, 12.7 ± 6.43) (Fig. 1B). Additionally, SUVmax was significantly lower in patients with exon 19 or exon 21 point mutations (median, 8.8; range, 0–38.2) than in patients with wild-type EGFR (*P* < 0.001; median, 11.8; range, 0–39.3). No statistically significant correlation was found between the patients with exon 18 or exon 20 point mutations and those with wild-type EGFR (*P* = 0.053) because the number of cases was too few. However, there was no significant difference in the SUVmax among different kinds of mutation in EGFR (exon 18, 12.2 ± 1.41; exon19, 9.39 ± 6.03; exon20, 8.57 ± 2.97; exon21, 9.18 ± 5.56, *P* = 0.807). For additional analyses, a lower SUVmax was associated with EGFR mutations in nonsmoking patients (mean, 9.09 ± 5.69 vs. 11.2 ± 6.13, *P* = 0.027) and in smoking patients (mean, 10.52 ± 5.83 vs. 13.39 ± 6.48, *P* = 0.022; Fig. 1C). With increasing SUVmax of the primary tumor, there was a trend toward decreasing incidence of EGFR mutations (*P* < 0.001) (Fig. 1D).

**Figure 2:**
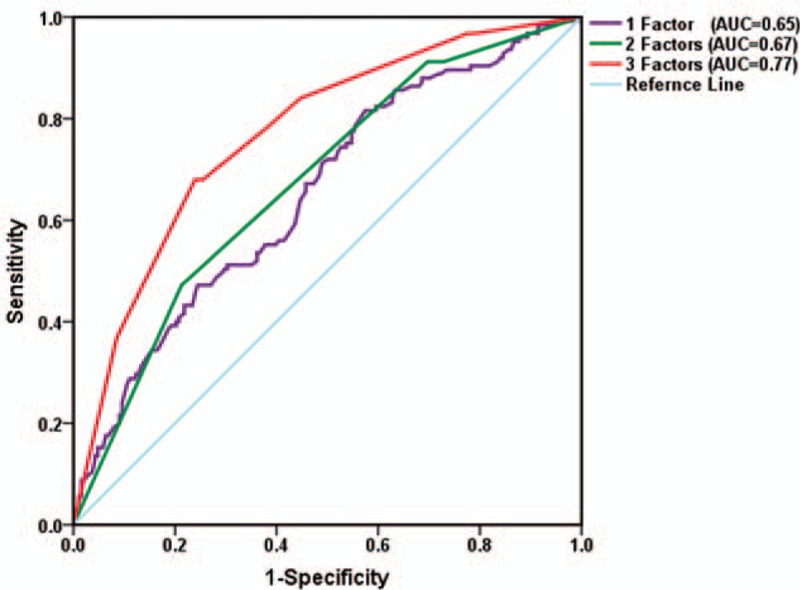
The receiver-operating characteristic (ROC) curve of the development cohort. Comparative ROC curves for 1 factor (maximum standardized uptake value [SUVmax]), 2 factors (SUVmax and primary tumor size), and 3 factors (SUVmax, primary tumor size, and smoking history) for predicting epidermal growth factor receptor mutation status. AUC = area under the curve.

ROC curve analysis was performed to show that the SUVmax cutoff point was 8.1 (Table 1), and AUC was 0.65 (95% confidence interval [CI] 0.60–0.72) (Fig. 2). EGFR mutations were more frequent in patients with low SUVmax (≤8.1) than in those with high SUVmax (>8.1) (56.6% vs. 31.4%; *P* < 0.001).

### 3.3 Multivariate analysis of various predictive factors of EGFR mutation status

In univariate analysis, EGFR mutation status was significantly associated with sex, smoking status, tumor histology, tumor grade, primary tumor size, and SUVmax of the primary tumor (Table 1). In the multivariate analysis, smoking status (nonsmoking), primary tumor size (≤5 cm), and SUVmax (≤8.1) were the statistically significant predictors of EGFR mutations (Table 2). Sex, histopathology, and AJCC stage were not significant predictors. ROC curves were generated and analyzed to quantify the predictive value of these factors (Fig. 2). When these 3 parameters were considered, the AUC was remarkably increased to 0.77 (95% CI 0.72–0.82), with sensitivity equal to 68.0% (95% CI 59.1%–76.1%). Specificity was increased to 76.1% (95% CI 69.3%–82.0%), the positive predictive value was increased to 65.4% (95% CI 52.2%–81.3%), and the negative predictive value increased to 78.1% (95% CI 66.4%–89.7%). Interestingly, 93.33% of patients who were smokers and had SUVmax >13.5 and primary tumor size >5 cm had wild-type EGFR (Supplemental Digital Content 2).

**Table 2:**
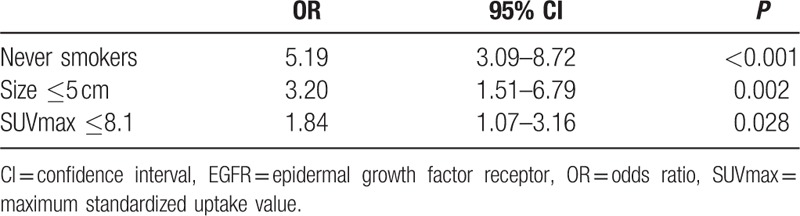
Multivariate analysis for various predictive factors of EGFR mutation.

### 3.4 Validation of predictions of EGFR mutation status

We next considered an independent, prospective human NSCLC dataset to validate our predictive model. The validation cohort included 85 patients, and the essential characteristics of the patients are shown in Table 3. Similar to the training set, all of the patients had undergone EGFR gene testing and PET/CT scans before therapy. The median age of the patients was 62 years (range 29–81), 75.3% were male, and 58.8% were smokers. The primary tumor size was ≤5 cm maximum diameter in 61 patients (71.8%). The SUVmax of 25 patients was ≤8.1.

**Table 3:**
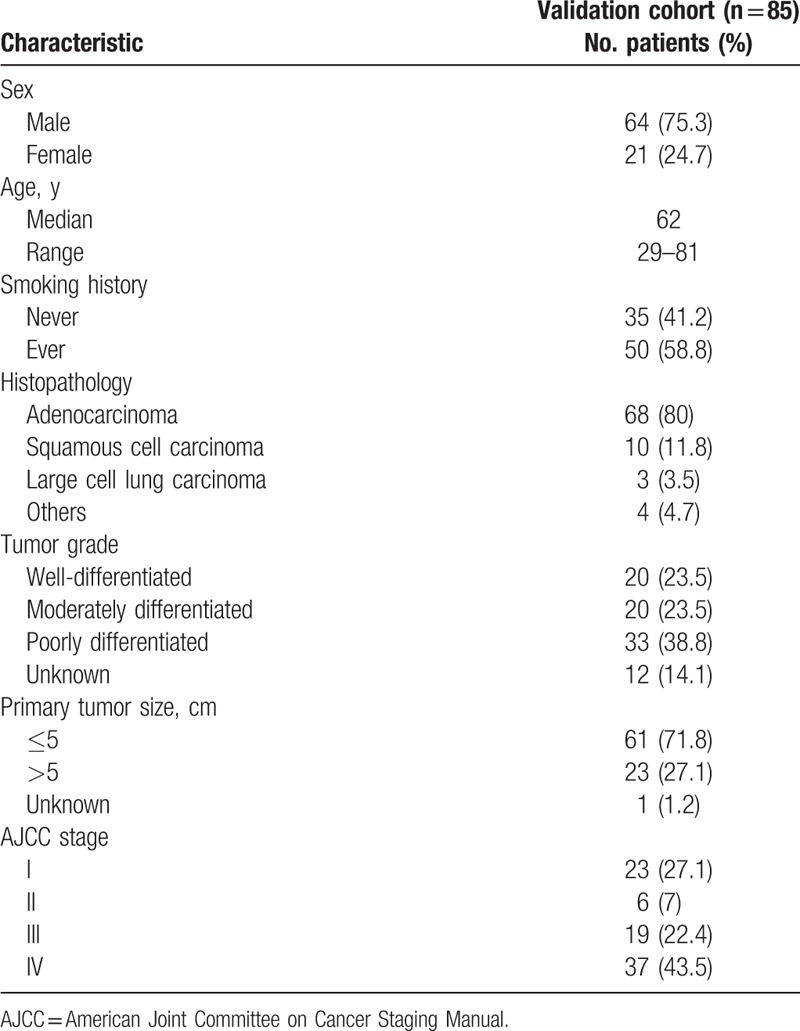
Patient characteristics of validation cohorts.

**Figure 3:**
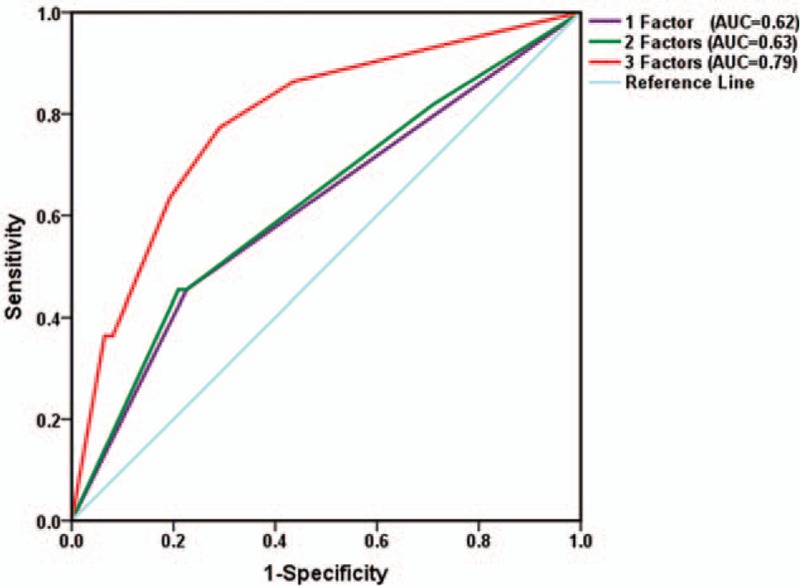
The receiver-operating characteristic (ROC) curve of the validation cohort. Comparative ROC curves for one factor (maximum standardized uptake value [SUVmax]), 2 factors (SUVmax and primary tumor size), and 3 factors (SUVmax, primary tumor size, and smoking history) for predicting epidermal growth factor receptor mutation status. AUC = area under the curve.

The AUC for the model was 0.79 (95% CI 0.69–0.90). As for the development dataset, the predictive ability was better with 3 parameters than with 2 parameters or 1 parameter (Fig. 3). The sensitivity, specificity, and overall accuracy were 72.7% (95% CI 49.8–89.3), 76.2% (95% CI 63.8–86.0), and 75.3%, respectively (Supplemental Digital Content 3).

## 4 Discussion

In this study, we retrospectively assessed the relationship between FDG uptake and EGFR mutation status in NSCLC patients. After that, we established a predictive model for EGFR status by combining low SUVmax (≤8.1), nonsmoking status, and primary tumor size (≤5 cm) and validated the model by considering a prospective cohort.

There is conflicting information about the relationship between SUVmax and EGFR mutation status in previous studies. Na et al^[14]^ showed that patients with low SUVmax (<9.2) in primary tumors were likely to have EGFR mutations, and Mak et al^[15]^ suggested that normalized SUV >5, defined as the ratio of SUVmax to the SUV of blood in the pulmonary artery, might be associated with wild-type EGFR genotype. Similar to Na et al's and Mak et al's study, our findings show that a lower SUVmax (≤8.1) is linked to EGFR mutations. However, Huang et al^[16]^ showed that patients with SUVmax ≥9.5 were more likely to have EGFR mutations than those with SUVmax <9.5, and Ko et al^[17]^ suggested that a higher SUVmax (≥6) is associated with EGFR mutations.

The possible reasons for the different results between our study and those 2 previous studies are as follows: Huang et al and Ko et al only enrolled 77 patients and 132 patients, respectively, and the histological types were all adenocarcinoma. Patients with the bronchioloalveolar subtype of adenocarcinoma were excluded in the study of Huang et al. In our study, the bronchioloalveolar subtype was associated with EGFR mutations and low SUVmax. Otherwise, pathological samples were mostly obtained via biopsy or pleural effusion cell blocks in the studies of Huang et al and Ko et al (88% and 89.4%, respectively). Some patients with EGFR mutations may have been mistaken for patients with wild-type EGFR because of tumor heterogeneity. In addition, Huang et al only enrolled advanced lung adenocarcinoma patients, and most patients were at stage IV (81%). Our results suggested that stage IV was associated with EGFR mutations, and this result explains why the study of Huang et al had a high proportion of EGFR mutations. Tumors with greatest dimension <1 cm were excluded in the study of Ko et al, but our results suggest that small tumor volumes are associated with EGFR mutations. These factors may explain the discrepant results for SUVmax in our study.

However, different from above researches, Lee et al showed that low SUVmax was significantly related to the EGFR mutations in univariate analyses, but not in multivariate analyses. As similar with our study, tumor size was associated with the EGFR mutation status in this research. We note that most of patients (67.0%) were stage IV in the study, though the enrolled patients were 206. Compared with this, there were 316 patients in our study, and 49.4% patients were stage IV. The clinical stage was excluded from our predictive model in multivariate analyses, suggesting that clinical stage may affect the relationship between SUVmax and EGFR mutation status. This maybe the reason why SUVmax was significantly associated with the EGFR mutation status in the study of Lee et al.^[18]^ This should be confirmed by further prospective study. In a word, low SUVmax may be associated with the EGFR mutation status in NSCLC patients.

In the present study, there is a strong negative correlation between primary tumor size and EGFR mutations. Large primary tumor size (≥5 cm) is significantly associated with wild-type EGFR. We also found that high SUV is associated with large primary tumor size (≥5 cm). In addition, FDG uptake has been shown to be associated with tumor proliferation in NSCLC.^[19–21]^ Hence, wild-type EGFR may be associated with invasive tumors.

After analyzing the relationships between EGFR mutation status, SUVmax, and clinical features, we established a predictive model by using retrospective analysis, and we validated by prospective analysis for estimating the EGFR mutation status in patients. The results from the development cohort show that the model has moderate accuracy and higher predictive value in patients with EGFR mutations than in those with wild-type EGFR. We note that the incidence of EGFR mutations in the development cohort is higher than in the validation cohort (39.9% vs. 25.9%). One reason for this may be selection bias in the validation cohort. Additionally, the incidence of smoking in patients is different between the development cohort (51.3%) and the validation cohort (59.2%). However, although the incidence of EGFR mutations differs between these 2 groups, the accuracy in the validation cohort (75.3%) is similar to the accuracy in the development cohort (72.8%). This also suggests that the predictive model is reliable.

The predictive model does not require distinguishing between squamous carcinoma or nonsquamous cell cancer. One of the significant advantages of the present study is that reliable criteria for predicting EGFR mutations were established using clinical and imaging parameters: nonsmoker status, low SUVmax, and small primary tumor size (≤5 cm), based on the outcomes of the multivariate analysis. The model was validated, and the accuracy was acceptable.

There are several limitations to our study. The FDG uptake is affected by many factors, such as attenuation correction, time of SUV evaluation, and reconstruction method and parameters for the scanner. Different SUV cutoff values may thus be obtained at different institutions. Furthermore, we have to realize that tissue testing is the criterion standard for judging EGFR mutation status. If a patient does not respond well to other treatments and cannot undergo gene testing, the predictive model might be helpful for assessing EGFR mutation status. This may determine whether to use EGFR-TKI or not.

## 5 Conclusion

In conclusion, the results of the present study, which were validated by a prospective sample, demonstrate that EGFR mutation status is associated with FDG uptake, smoking history, and primary tumor size in NSCLC patients. The model can be efficien0t for determining EGFR mutation status to estimate the combined effects of these 3 factors, especially when genetic testing is not practical or when there is insufficient tumor tissue. However, for further substantiation of the current results, a large prospective study is warranted.

## Supplementary Material

Supplemental Digital Content
