# Supplementary material for: 18F-FDG uptake for prediction EGFR mutation status in non-small cell lung cancer
Source: Medicine (Baltimore). 2016 Jul 29;95(30):e4421. doi: 10.1097/MD.0000000000004421 (PMC5265876; doi:10.1097/MD.0000000000004421)
Supplement: Supplemental Digital Content [file medi-95-e4421-s001.doc]

Supplemental Digital Content 1. Figure: Patients included in18F-FDG PET/CT analysis





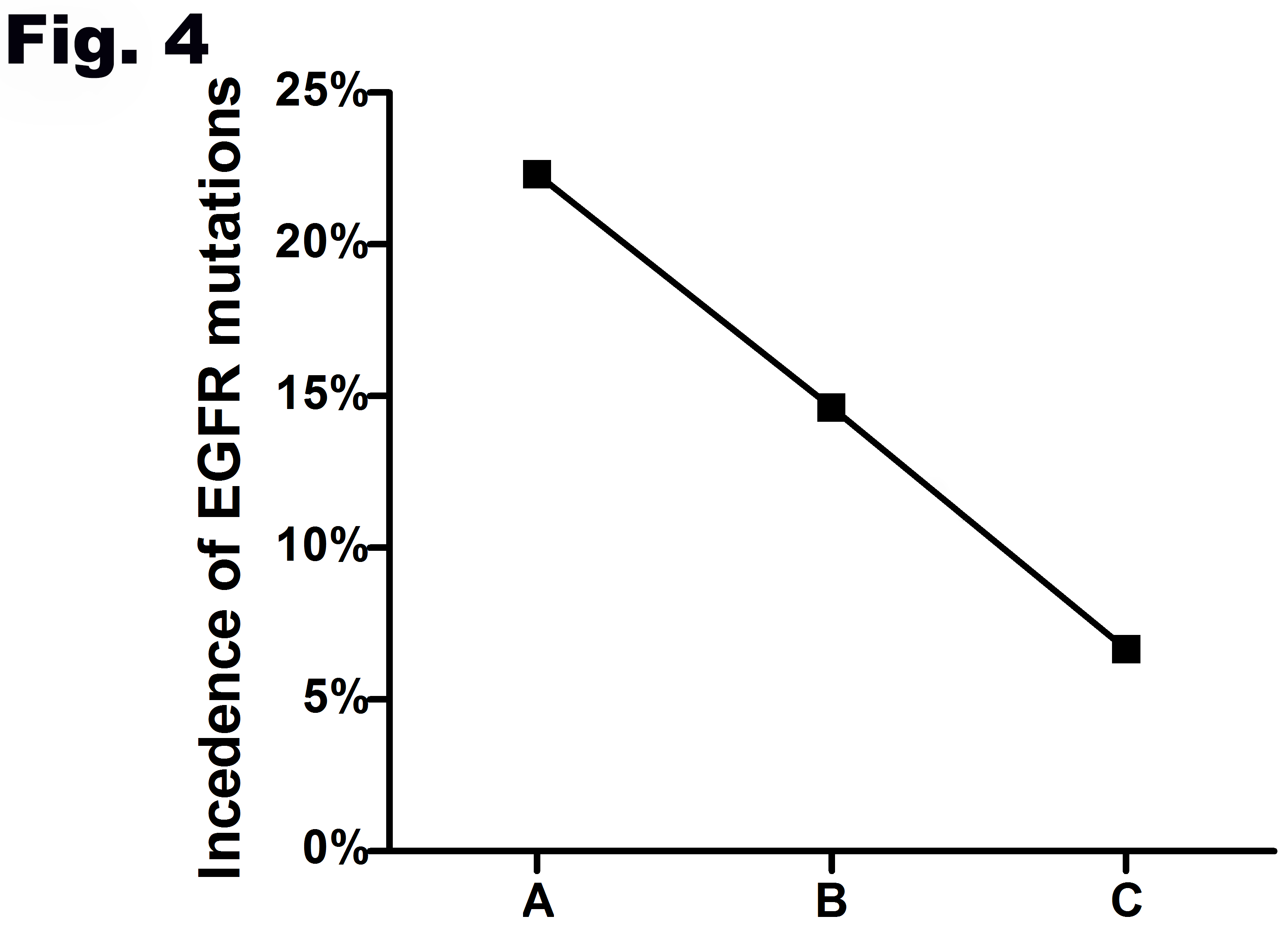


Supplemental Digital Content 2. Figure: The incidence of wild-type EGFR in different patient subgroups. A: SUVmax > 13.5; B: SUVmax > 13.5 and primary tumor size > 5 cm; C: SUVmax > 13.5, primary tumor size > 5 cm and history of smoking

| **Table 4.** Characteristics of predictive and validation models | | |
| --- | --- | --- |
|  | The development cohort | The validation cohort |
| sensitivity | | |
| % | 68.0 | 72.7 |
| 95% CI | 59.1 to 76.1 | 49.8 to 89.3 |
| specificity | | |
| % | 76.1 | 76.2 |
| 95% CI | 69.3 to 82.0 | 63.8 to 86.0 |
| positive predictive value | | |
| % | 65.4 | 51.6 |
| negative predictive value | | |
| % | 78.1 | 88.9 |
| Overall accuracy | 72.8 | 75.3 |
